# A Brief Overview of lncRNAs in Endothelial Dysfunction-Associated Diseases: From Discovery to Characterization

**DOI:** 10.3390/epigenomes3030020

**Published:** 2019-09-13

**Authors:** Rashidul Islam, Christopher Lai

**Affiliations:** 1 Department of Health Technology and Informatics, Hong Kong Polytechnic University, Hong Kong, China; rashidul.islam@studenti.unicam.it; 2 Health and Social Sciences Cluster, Singapore Institute of Technology, Singapore 138683, Singapore

**Keywords:** endothelial dysfunction, lncRNAs, angiogenesis, hypertension, atherosclerosis, diabetes

## Abstract

Long non-coding RNAs (lncRNAs) are a novel class of regulatory RNA molecules and they are involved in many biological processes and disease developments. Several unique features of lncRNAs have been identified, such as tissue-and/or cell-specific expression pattern, which suggest that they could be potential candidates for therapeutic and diagnostic applications. More recently, the scope of lncRNA studies has been extended to endothelial biology research. Many of lncRNAs were found to be critically involved in the regulation of endothelial function and its associated disease progression. An improved understanding of endothelial biology can thus facilitate the discovery of novel biomarkers and therapeutic targets for endothelial dysfunction-associated diseases, such as abnormal angiogenesis, hypertension, diabetes, and atherosclerosis. Nevertheless, the underlying mechanism of lncRNA remains undefined in previous published studies. Therefore, in this review, we aimed to discuss the current methodologies for discovering and investigating the functions of lncRNAs and, in particular, to address the functions of selected lncRNAs in endothelial dysfunction-associated diseases.

## 1. Introduction

The endothelium, which is also known as the “tissue-blood barrier”, is a monolayer of endothelial cells (ECs) that lines on the outer surface of blood vessels. This monolayer covers approximately 400 m^2^ throughout the entire blood circulation [1,2], and it was previously considered as merely a barrier with no specific function, but permeable to electrolytes and water [3]. Over time, the regulatory functions of the endothelium have gradually discovered. These include the control of vascular tone, cellular adhesion, blood fluidity, thrombosis, homeostasis, smooth muscle cell proliferation, angiogenesis, metabolism, and vascular permeability. They are facilitated by the synthetization and release of different important molecules, such as nitric oxide (NO), prostacyclin (PGI2), thromboxane (TXA2), bradykinin, endothelium derived hyperpolarizing factor (EDHF), endothelin-1 (ET-1), thromboxane A2, prostaglandin H2 (PGH2), and reactive oxygen species (ROS) [4,5,6]. The endothelium also actively maintains a precise balance in vasodilators, vasoconstrictors, and adhesion molecules for auto-regulation [7]. 

NO is considered to be the most potent endogenous vasodilator in our body. NO is synthesized from l-arginine metabolism by endothelial NO synthase (eNOS) [5] and then diffuses to vascular smooth muscle cells (VSMCs). The latter subsequently activates soluble guanylyl cyclase (sGC) and stimulates the production of cyclic guanosine 3′,5′-monophosphate (cGMP) from guanosine triphosphate (GTP). Finally, the intracellular Ca^2+^ concentration is decreased by cGMP through inhibiting phosphorylation pathways that enhances SMC relaxation and vascular tone [8]. 

The disruptions of the auto-regulatory processes in endothelium, including decreased production and bioavailability of NO, impaired endothelial dependent-NO-mediated vascular relaxation, upregulated inflammatory genes expression level, dysregulated hemodynamic, adhesion molecules up-expression, excessive ROS production, and enhanced vascular endothelium permeability [3,4,5,6,7,8]. They are conclusively referred as “endothelial dysfunction (ED)”. Subsequently, the dysfunction leads to the development and progression of different forms of vascular diseases [6]. Numerous studies have discovered significant correlations between ED and vascular-mediated diseases, such as angiogenesis, atherosclerosis, hypertension, diabetes, insulin resistance, obesity, etc. [6]. However, the underlying pathophysiology of ED and the correlations between functional roles of lncRNAs and vascular-mediated diseases remain unclear. Therefore, we aim to provide a brief playbook for the discovery and characterization of lncRNAs in ED-associated diseases and, in particular, to provide a concise overview of current knowledge on lncRNAs in ED-associated diseases. It is believed that with a better understanding the correlations of lncRNAs and ED-associated diseases, researches can make a positive impact on the development of novel biomarkers and therapeutic targets in ED-associated disease treatments.

## 2. lncRNAs in the Regulation of Gene Expression 

Advanced genome- and transcriptome-wide analyses discovered that <2% of human genome contains protein coding transcripts, while >75% of which were transcribed into noncoding RNAs (ncRNAs) and have no protein coding potentiality [9,10]. The ncRNAs can be broadly classified into two groups based upon their sizes: the short noncoding RNAs which are less than 200 nucleotides (nt) in length, such as small interfering RNAs (siRNAs), small nuclear RNAs (snRNAs), small nucleolar RNAs (snoRNAs), PIWI-interacting RNAs (piRNAs), and microRNAs (miRNAs), etc., and the lncRNAs, which are more than 200 nt in size [11]. Both ncRNAs classes were found to be involved in many pathophysiological processes [12], but only the short ncRNAs, especially miRNAs, were extensively studied. miRNAs predominate the RNA biology research due to the higher sequence conservation across multiple species and better understanding of mechanism in every step of biological processes [13]. 

Over the last decades, lncRNAs have received much attention by researchers because of their gradually disclosed biological functions. Although they were found to be involved in a range of developmental processes and diseases, the underlying pathophysiological mechanisms remain limited. Despite there being controversial findings that were reported in previous lncRNAs studies [14,15,16], the functions of many lncRNAs are largely unclear. Those findings on lncRNAs have indeed provided important evidence regarding their roles in the biological regulation. 

Similar to mRNA, the mechanisms of biogenesis of lncRNAs are also gradually uncovered. Nowadays, most of the lncRNAs are usually transcribed by RNA polymerase-II (pol II), and then capped at 5′-end, spliced and polyadenylated at 3′-end [17]. lncRNAs are distributed throughout the genome. Based on their genomic locations and characteristics, they can be categorized into five types: sense, antisense, bidirectional, intronic, or intergenic. The details on genomic locations of lncRNAs refer to Laurent et al. 2015 [18]. More importantly, as far as molecular functions of lncRNAs are of concerned, numerous studies revealed that their cellular localization is the primary determinant of their functions [19,20,21]. A number of studies were conducted by Batista and Chang, 2013, Mercer et al. 2008 and others to detect the subcellular localizations of lncRNAs [20,21]. In addition, the traits of lncRNAs, presented in nucleus and/or cytoplasm. Interestingly, the cytoplasmic lncRNAs are mainly involved in the regulation of translation or signal transduction process. On the other hand, the nuclear lncRNAs are mainly implicated in the regulation of gene transcription process [21,22]. As mentioned earlier, thousands of lncRNAs were found to be involved in various pathophysiological processes, including ED and its associated diseases. Unfortunately, only a few identified lncRNAs were functionally characterized by previous published studies [12,13,23,24].

lncRNAs can regulate gene expression at both the transcriptional and post-transcriptional levels through diverse mechanisms [25,26]. In most cases, lncRNAs can regulate the transcription process via chromatin modulation [23]. lncRNAs can also bind single or multiple chromatin modifying complexes and target their activities to particular DNA loci. For example, HOX transcript antisense RNA (HOTAIR) was found to be directly associated with Lys-specific demethylase 1 (LSD1), Polycomb repressive complex (PRC2), of which are responsible for removing the active demethylation of lysine 4 residue on histone 3 (H3K4me2) [24]. Another lncRNA antisense transcript of GATA6 (GATA-AS) was found to regulate endothelial gene expression through interacting with epigenetic regulator lysyl oxidase-like 2 (LOX2) to remove activated trimethylation of lysine 4 of histone H3 (H3K4me3) [27]. 

On the other hand, lncRNAs-mediated chromatin modulations can either activate or repress gene expression, depending on the nature of the enzyme or factors to be bound during remodeling [23,28,29]. Furthermore, it is also revealed that the carboxyl-teminal domain (CTD) of Pol II can bind with chromatin modifying complexes and alter lncRNAs transcription [23,30]. In human umbilical vein endothelial cells (HUVECs), MANTIS has been found to act as a scaffolding lncRNA within a chromatin remodeling complex and up-regulate endothelial genes, including SRY (Sex Determining Region Y)-Box 18 (SOX18), mothers against decapentaplegic homologue 6 (SMAD6), and chicken ovalbumin upstream promoter transcription-factor-2 (COUP-TFII) transcription by enabling pol II binding to the transcription stat sites (TSS) [31]. However, lncRNAs transcription can cause chromatin remodeling and it leads to either stimulating or inhibiting the binding of regulatory factors [32,33], and even directly influence the transcription process. For example, several lncRNAs synthesized from arthrobacter luteus (Alu) short interspersed elements (SINE) elements and directly bound with pol II can repress the transcription of specific mRNA during heat shock [34]. Furthermore, the assembly of pre-initiation complex *in trans* can be inhibited by highly up-regulated lncRNAs through the formation of a DNA-RNA triplex structure [35]. Importantly, lncRNAs can fold into structures that stimulate or inhibit the activity of different transcription factors and mimic DNA-binding sites [23]. For example, growth arrest specific 5 (GAS5) represses glucocorticoid receptor (GR)-mediated transcription by folding into a particular shape that mimics the DNA-binding site of the GR [36]. Besides, lncRNAs can also regulate gene expression through binding particular transcriptional factors to inhibit the nuclear localization of specific transcription factors [37]. For instance, the non-coding repressor of NFAT (NRON) obstructs nucleocytoplasmic shuttling of the transcription factor nuclear factor of activated T-cells (NFAT) and indirectly represses the transcription process [37]. A recent study has described an atlas of transcriptional regulating lncRNAs in HUVECs under hypoxic condition, which suggests the potential of lncRNAs as a future development biomarker of endothelial hypoxia in atherosclerosis lesion and/or other vascular diseases [38]. 

Besides, lncRNAs are also critically involved in the mRNA processing and post-transcriptional regulation by serving as promising source of miRNA, negative regulator of miRNA and stimulator of miRNA-independent mRNA degradation [30]. In addition, antisense lncRNAs can also directly modulate mRNA expression via a close association of the duplex with adenosine deaminase acting on RNA (ADAR) enzymes in double-stranded RNA [39]. Furthermore, lncRNAs are also contributed in the post-transcriptional events and protein synthesis. For example, lncRNA containing SINEB2 repeat elements were associated with 5′ region of mRNA to upregulate the translational process [40]. Likewise, lncRNAs are implicated in regulating mRNA stability, e.g., BACE1 (beta-secretase 1)-antisense RNA (BACE1AS) increases BACE1 mRNA stability through the abrogation of miRNA-induce repression [41]. In contrast, lncRNAs can mask the miRNA-binding sites on a specific target mRNA in order to block miRNA-induced silencing by generating the RNA-induce silencing complex [42]. In addition, linear or circular lncRNAs can function as miRNA decoys to sequester miRNAs from their target mRNAs [43]. 

There are several types of lncRNAs that have been characterized as a post-transcriptional regulator of endothelial genes. For example, antisense mRNA (sONE) was found to mediate the post-transcriptional down-regulation of eNOS during hypoxia. It plays a critical role in the etiology of vascular diseases, including pulmonary arterial hypertension [44,45]. In summary, lncRNAs might directly influence the expression level of endothelial genes at the transcription and/or post-transcriptional level, leading to impaired endothelium function in both physiological and pathological conditions. 

## 3. Functional Roles of lncRNAs in Endothelial Dysfunction

Impaired endothelial function is linked with the initiation and progression of various diseases, including abnormal angiogenesis, atherosclerosis, hypertension, and diabetes. ED can be triggered by different biochemical and biomechanical stimuli, such as modified low-density lipoprotein (LDL), Interleukin 1 beta (IL-1β), disturbed blood flow, etc., of which have been considered as the primitive step to induce atherosclerotic lesion [46]. Similarly, the synthetization and expression of adhesion molecules and chemokines, such as vascular cell adhesion molecule-1 (VCAM-1), E-selectin, monocyte chemoattractant protein-1 (MCP-1), and C-X3-C motif chemokine ligand 1 (CX3CL1), can accelerate leukocyte adhesion. It subsets onto the vessel wall and compromises the endothelial integrity, leading to the initiation of vascular inflammation and atherosclerosis [47,48]. Although the regulatory mechanisms of ED and its associated diseases have been identified, their underlying mechanisms are under further investigation for improving the diagnostic and treatment accuracy. 

Numerous lncRNAs are functionally correlated with ED and its associated diseases in recent studies, such as highly upregulated in liver cancer (HULC) [49,50], metastasis associated lung adenocarcinoma transcript 1 (MALAT1) [51,52,53,54,55,56,57,58,59,60], maternally expressed 3 (MEG3) [61,62,63,64,65,66,67,68], taurine up-regulated 1 (TUG1) [69,70,71], plasmacytoma variant translocation 1 (PVT1) [72,73], ANRIL (CDKN2B-AS1) [74,75], HOTAIR [76,77], retinal non-coding RNA3 (RNCR3) [78,79], X-inactive specific transcript (XIST) [80], and H19 imprinted maternally expressed transcript (H19) [81]. Moreover, lncRNAs can interact with different functional molecules and alter the indispensable steps of ED, including NO production, vascular tone regulation, endothelium activation/deactivation, inflammatory response, barrier function, EC proliferation, migration, and apoptosis [82]. For example, one of the most widely studied lncRNAs, MALAT1, was involved in the regulation of EC proliferation, apoptosis through inhibiting cell cycle progression, and modulation in the expression level of vascular endothelial growth factor (VEGF) [55,56,59]. Additionally, the knockdown of MALAT1 can reduce the S-phase cyclins named cyclin A2 (CCNA2), cyclin B1 (CCNB1), and cyclin B2 (CCNB2) in ECs [59], decrease the expression level of phosphorylated P^38^ in retinal ECs [55], and reduce the activation of phosphatidylinositol 3-kinase (PI3K) and AKT serine/threonine kinase 1 (Akt) phosphorylation in human brain vascular endothelial cells (VECs) [60]. It further increases the expression level of cell cycle inhibitory genes P^21^, P^27Kip1^, interleukin 6 (IL-6), and tumor necrosis factor α (TNFα) in HUVECs, leading to subsequent decreased cell proliferation and increased cell apoptosis [56,60]. Besides, overexpressed MALAT1 promotes EC proliferation and inhibits cell apoptosis [60]. Moreover, the pharmacological inhibition of MALAT1 expression in vivo could reduce blood vessel capillary density and blood flow recovery, which suggests its involvement in EC proliferation and vascular outgrowth in in vitro condition [59]. Its interactions with different miRNAs (miR-22-3p, miR-320a, miR-26b) and modulate EC homeostasis, proliferation, apoptosis, autophagy, angiogenesis, and vascular inflammation were further revealed [58,83,84]. 

**Figure 1:**
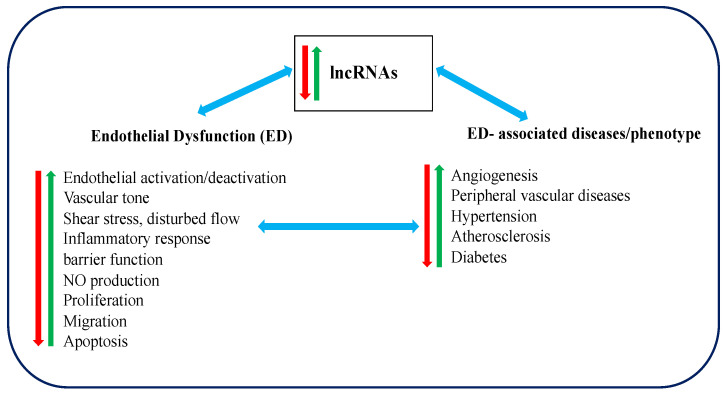
A summary of the functional correlations between Long non-coding RNAs (lncRNAs), Endothelial dysfunction (ED) and ED-associated diseases (red arrows represent downregulation or inhibition, green arrows represent upregulation and blue arrows represent possessing causal relationship).

On the other hand, another widely studied lncRNA, MEG3, was found to be downregulated in EC. The knockdown of MEG3 could increase EC proliferation, migration, and angiogenesis through regulating the PI3K/Akt singling pathway [63,67]. Similar to MALAT1, MEG3 can also interact with miR-9 and modulate EC proliferation and angiogenesis [63]. Taking altogether, it can be postulated that lncRNAs play a significant role in development and progression of ED and its associated diseases. Figure 1 presents a summary of the known functional correlations between lncRNAs, ED and ED associated diseases such as irregular angiogenesis, vascular diseases, hypertension, diabetes, and atherosclerosis.

## 4. Methods for Studying lncRNA: From Discovery to Function

**Figure 2:**
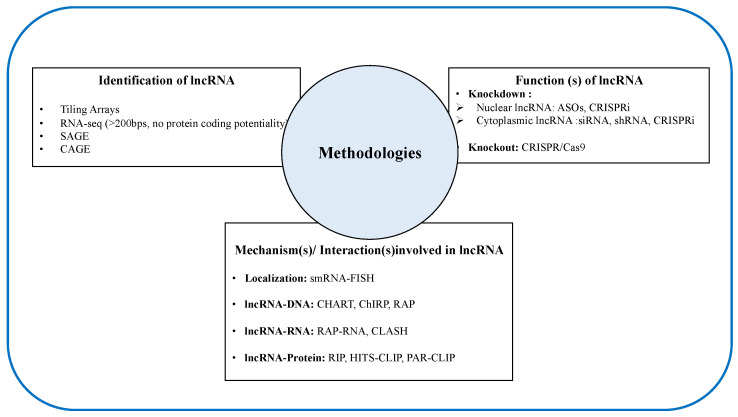
A summary of the current available methodologies for the discovery and characterization of the cellular functions of lncRNAs. RNA-seq: RNA sequencing; SAGE: Serial analysis of gene expression; CAGE: Cap analysis of gene expression; ASOs: Antisense oligonucleotides; CRISPRi: CRISPR interference; siRNA: Small interfering RNA; shRNA: Short hairpin RNA; smRNA: Single-molecule RNA; FISH: Fluorescence in situ hybridization; CHART: Capture hybridization analysis of RNA target; ChIRP: Chromatin isolation by RNA purification; RAP: RNA antisense purification; CLASH: Cross-linking, ligation and sequencing of hybrid; RIP: RNA immunoprecipitation; HITS-CLIP: High-throughput sequencing of RNA isolated by cross-linking immunoprecipitatio; PAR-CLIP: Photoactivatable ribonucleotide-enhanced cross linking and immunoprecipitation.

Unlike mRNA, the expression levels of lncRNAs genes are relatively lower and rather cell-and/or-tissue-specific. Additionally, the sequence of lncRNA does not provide much information about their functional information, making the discovery and characterization of lncRNA very challenging [85,86]. In this chapter, we attempted to briefly summarize the current available methodologies (Figure 2) for the discovery and characterization of cellular functions of lncRNAs.

### 4.1. Methods to Identify/Discover lncRNAs

A lncRNA study usually begins with the identification process. As mentioned earlier, lncRNAs are tissue specific and they have a lower expression level than coding genes, therefore it is always very critical and challenging to select the most appropriate method in the identification process [87]. Currently, there are several advanced techniques that are available to identify lncRNAs (Table 1), and these advanced techniques will be briefly described in the following paragraphs:

#### 4.1.1. Tiling Arrays

Tiling arrays contain high-density oligonucleotide probes that can encompass non-repetitive sequence of specific chromosome and whole genome analysis [88,89]. The advantage of using tiling arrays over the conventional microarray method is that the former allows alternative splicing analysis, polymorphism detection and identification of novel transcripts [86,88,89]. For example, HOTAIR was identified from the human HOX gene cluster by using high resolution tiling array that was designed with 400 thousands of overlapping probes [100]. Although tiling arrays have been considered as the gold standard method for detecting and quantifying the expression analysis of transcripts, it is now largely replaced by next generation sequencing (NGS) technologies, owing to its higher background noise and higher cost [86,101]. 

#### 4.1.2. Serial Analysis of Gene Expression (SAGE)

SAGE is one of the first genome-wide gene expression analyzing methods that allows for comprehensive, unbiased, and quantitative analysis of gene expression profiles without prior knowledge of gene of interest [90,91]. For example, a systemized 272 human SAGE experiments were performed to identify and analyze the expression profile of lncRNAs in both normal and cancerous tissues [102]. 

#### 4.1.3. Cap Analysis of Gene Expression (CAGE)

CAGE is a high-throughput NGS based method that allows for the identification and quantification of the expression of 5′-capped RNAs [92,93,94,95]. In contrast, SAGE can only cover the 3′-end of RNAs and it is unable to provide any information about 5′-end regulatory elements [103]. A total of 19,175 lncRNAs were functionally identified and profiled through integrating various CAGE-seq data analysis [104]. Despite having the aforementioned advantages over SAGE and hybridization based methods, CAGE itself is limited by the 5′-capped transcripts and it is unable to cover circular RNAs together with non-caped RNAs [93,105].

#### 4.1.4. RNA Sequencing (RNA-Seq)

RNA-seq has competence in detecting sequence variations, alternative splicing isoforms, gene fusion events, and novel splice junctions over using hybridization or sanger sequencing based methods e.g., Tiling arrays, SAGE, and CAGE etc. [97,98,106,107]. In RNA-seq, non-polyadenylated ((poly(A)-) RNAs are first depleted and poly(A)+ RNAs are converted into the cDNA fragments library. Subsequently, sequencing adaptors are introduced into each cDNA fragment and short sequencing is acquired by any high-throughput sequencing technologies [97,108,109]. Finally, sequencing reads align with the reference genome and that will provide genome-wide transcription mapping. The library preparation and final bioinformatics analysis are the major challenges and drawbacks of RNA-seq despite having numerous advantages.

### 4.2. Methods to Study the Functional Roles of lncRNAs

Following the identification step, the second challenge in both in vitro and in vivo studies is to investigate whether the newly identified lncRNAs are involved in any biological role or not. Nowadays, there are several methods to unveil the functions of lncRNAs and to classify lncRNAs into different biological phenotypes [110,111]. Table 2 summarizes a brief overview with a comparison of the advantages and disadvantages of the widely used methods for investigating lncRNAs cellular functions under different physiological and/or pathological conditions.

#### 4.2.1. RNA Interference (RNAi)

RNAi is the most extensively used method for achieving “loss-of-function study” through the knockdown of lncRNAs [111,112,113,127]. RNAi methods, such as small interfering RNA (siRNA) and short hairpin RNA (shRNA), usually use 20–40 nucleotide transcripts which are complimentary to the target transcript and make duplexes that are subsequently degraded by the cellular machinery [112]. siRNAs target the transcript of interest in a transient fashion, while shRNAs are stably expressed [113]. It is still controversial that RNAi methods can only target cytoplasmic lncRNAs but not nuclear lncRNAs [19,128]. Nevertheless, recently, the RNAi method has been effectively used to target some nuclear lncRNAs [113,129]. It has been suggested that the complex structured nature and the lower expression level of lncRNAs can hinder the accuracy of RNAi methods. Therefore, further modification procedures are needed to address these limitations [87,115].

#### 4.2.2. Antisense Oligonucleotides (ASOs)

ASOs are synthetic single stranded nucleic acid derivatives, forming RNA-DNA hybrids and inducing the cellular RNAs degradation by endogenous RNase H [116,117,118,130]. The knockdown efficiency of both RNAi and ASOs critically depends on the subcellular localization of lncRNAs. For example, the RNAi methods effectively suppress cytoplasmic lncRNAs, but nuclear lncRNAs are more efficiently suppressed by ASOs [119]. Therefore, it has been recommended that the RNAi and ASOs can be used together to target dual-localize (present in both nucleus and cytoplasm) lncRNAs [119]. Today, ASOs based method is considered as a promising tool for investigating lncRNAs functionality, especially for novel lncRNAs.

#### 4.2.3. CRISPR/Cas System

The CRISPR/Cas system is the most advanced, robust, and highly efficient gene manipulating method for revealing the biological functions of lncRNAs, both in vitro and in vivo [120,131]. There are different manners of the CRISPR/Cas system that can be applied to induce a partial or complete deletion of the lncRNAs genomic locus [120,132], insert polyadenylated signals and make an interruption between the promoter and lncRNA sequence [133], target a transcriptional activator complex to the promoter [134], and insert a robust promoter upstream of the gene to overexpress lncRNA in order to accomplish successful “gain or loss-of-function” studies of lncRNAs [135]. The knockout of lncRNA study was initiated while using a pair of single guide RNA (sgRNA) that targeted two specific locations flanking the selected lncRNA gene and permanently eradicate the entire genomic locus [122]. Alternatively, lncRNA transcription can also be abolished by depleting the main promoter region of lncRNA of interest. It gives higher knockout efficiency than conventionally deleting whole genomic locus [120,123]. However, if lncRNA contains multiple promoters in their sequence, then these promoters needed to be examined to find the best knockout effect on the expression of lncRNA. Moreover, the CRISPR/Cas9 system was further utilized to improve the silencing efficiency of lncRNAs through inserting a PolyA-transcriptional terminator [124,136]. A biallelic PolyA signal can also be inserted into different sites using this method, such as at the site immediately after the promoter, at the first exon, or at the first intron of the targeted gene through CRISPR/Cas9 mediated homology direct repair (HDR) system to enhance the silencing efficiency [121,124,136]. However, the target gene transcription cannot be entirely blocked by this method. Therefore, this method would be the best choice for the functional study of the lncRNA genes, whose deletions or complete knockout could induce lethal phenotype.

#### 4.2.4. CRISPR Interference (CRISPRi)

CRISPRi can efficiently silence any gene transcription by sgRNA facilitated targeted recruitment of the nuclease-dead deactivated Cas9 (dCas9)-Kruppel-associated box (KRAB) repressor domain to the TSS [125,126,137,138,139,140]. CRISPRi can be applied to target any lncRNA gene, owing to its capacity to act in a range of 1kb around the TSS and block 23 bp of targeted sequence [139,141]. With the application to chromatin modification nearby the TSS and transcriptional road-blocking ability, CRISPRi also expended to unveil the potential functions of lncRNAs. For example, the roles of lncRNAs in the production of cis-and-trans-acting RNA transcript [29], enhancer like function, cis-mediated regulation of gene function while using the CRISPRi method were published [29,142,143,144]. The CRISPRi allows for the repression of a targeted locus without whole genome editing, and characterized by highly efficient without no-to-little off-target effects due to false dCas9 binding or accidental silencing of other nearby regulatory elements [122,126]. However, the effectiveness of using CRISPRi methods for lncRNA study is limited by the prerequisite knowledge regarding the location of the promoter or enhancer. In addition, re-confirmation of the observed specific regulatory elements solely regulates the transcription of selected lncRNA.

### 4.3. Different Methods to Unveil the Functional Mechanisms of lncRNAs

Prediction of lncRNAs functions is arduous, because their sequence does not provide much information regarding their function alike mRNA. It is generally accepted that the localization of lncRNAs within the cell is the primary determinant of their molecular functions [19]. Broadly, there are two approaches, named “absolute approach” and “relative approach”, that are commonly used to detect RNA localization [145]. The absolute localization refers to the ratio of molecules or mass of RNA between two compartments. There are several quantification methods, such as Image analysis ((e.g., fluorescence in situ hybridization (FISH), Single-cell RNA FISH, multiplexed error-robust fluorescence in situ hybridization (MERFISH)), Microarray (e.g., Fractionation), are commonly used to detect the absolute localization of lncRNA [145,146]. On the other hand, the relative method merely calculates the ratio of concentrations in the two compartments while using several sequencing based methods, such as RNA-seq (e.g., Fractionation, APEX-RIP, APEX-seq, etc.) [145]. Yet, most of the global subcellular RNA maps are based on relative localization.

Absolute and relative methods can both efficiently detect specific lncRNA localization in different organelles, including nucleus, nucleolus, nuclear lamina, nuclear pore, nucleoplasm, cytoplasm, cytoplasmic membrane, insoluble cytoplasmic fraction, chromatin, cytosol, mono-and polysomes, mitochondria, mitochondrial matrix, outer mitochondrial membrane, endoplasmic reticulum (ER), ER membrane, ER lumen etc. [145]. For the sake of easy understanding, all of these organelles are considered under nucleus and cytoplasm. So, it is usually described that lncRNAs are present in the nucleus or/and cytoplasm, which help to predict their (lncRNAs) possible functions. For example, cytoplasmic lncRNAs mainly regulate mRNA stability or translation and influence cellular signaling cascades, while nuclear lncRNAs are critically involved in the chromatin interaction, transcriptional regulation, and RNA processing [147,148]. For details on genomic location of lncRNAs refer to Carlevaro-Fita J, Johnson R. [145].

Following the successful identification of subcellular localization of lncRNA, the interactions between lncRNAs and other regulatory macromolecules, including DNA, RNA, and protein in different biological processes can be investigated while using the different advanced methods that are summarized in Table 3. In the following chapter, we will briefly describe the most advanced and widely used methods for detecting the subcellular localization of lncRNAs and their molecular interactions with DNA, RNA, and protein.

#### 4.3.1. Localization of lncRNA: Single-Molecule RNA Fluorescence In Situ Hybridization (smRNA-FISH)

RNA fluorescence in situ hybridization (RNA-FISH) is a conventional method that si commonly used to detect the subcellular localization of lncRNAs [146,174]. Despite being able to identify the subcellular location of lncRNA, RNA-FISH has several limitations, for example, relatively low sensitivity and limitation to cover the comparatively highly abundant lncRNAs in the cells only [85,174]. Therefore, smRNA-FISH was then developed to quantify the absolute level of lncRNAs and the subcellular localization of low-abundance lncRNAs [19,149,150,175]. Yet, smRNA-FISH still has several drawbacks, including off-target hybridization, which needed to be improved for better understanding the lncRNAs functions.

#### 4.3.2. Techniques for Investigating lncRNA-DNA Interaction

##### Capture Hybridization Analysis of RNA Target (CHART)

CHART is a powerful method for mapping the genome-wide binding profile of chromatin-associated RNAs [151]. It utilizes a short number of 22–28 nt complementary biotinylated oligonucleotides that allow for the detection of lncRNAs of interest and its associated genomic DNA and/or protein from formaldehyde cross-linked chromatin [151,152]. In this method, designed probe can only target the region of potential binding sites instead of covering the entire length of lncRNA to capture the target complexes, hence the background signals can be reduced [86]. Along with the several advantages, it also comes with some shortcomings, such as using inefficient cross-linking agent formaldehyde and being time consuming, as time for digestion is required.

##### Chromatin Isolation by RNA Purification (ChIRP)

ChIRP utilizes a combination of oligonucleotides-based RNA-centric pull-down method with high-throughput DNA sequencing to disclose the unbiased lncRNA-DNA-protein complex [154]. ChIRP is one of the most commonly used techniques for analyzing chromatin associated lncRNAs. For example, HOTAIR associated chromatin DNA region (GA-rich-DNA-motif to nucleate broad domains of polycomb occupancy and histone 3 lysine 27 trimethylation (H3K27me3)) was analyzed while using ChIRP [155]. Similarly, genomic occupancy of roX2 RNA and telomerase RNA component were also discovered by ChIRP-seq [155]. Despite having the capacity of analyzing the genome-wide lncRNA-DNA binding sites and off target effect reduction, ChIRP has several disadvantages, such as higher noise-to signal ratio [155,176].

##### RNA Antisense Purification (RAP)

RAP is an alternative method for mapping the interactions between lncRNAs and chromatin while using antisense biotinylated oligonucleotides mediated hybridization [156]. RAP utilizes larger antisense RNA probe that can cover the full length of interested when compared to other techniques like ChIRP. It provides higher binding affinity and reduces the signal-to-noise ratio [86,157]. For example, RAP method was used to identified Xist binding site on X-chromosome [157].

#### 4.3.3. Techniques for Investigating lncRNA-RNA Interaction

##### RNA Antisense purification Followed by RNA Sequencing (RAP-RNA)

RAP-RNA is an improved version of RAP technique that can be used to identify RNA-RNA interactions by utilizing different cross-linking methods. There are three versions of the RAP-RNA method available: RAP-RNA^[AMT]^, RAP-RNA^[FA]^, and RAP-RNA^[FA-DSG]^ for analyzing the RNA-RNA interactions [158]. For example, RAP-RNA^[AMT]^ is commonly used to detect the RNA-RNA interactions (those RNAs are directly binding each other exclusive of protein intermediate) by generating aminomethyltrioxalen (AMT) mediated specific uridine bases cross-links [158]. Instead, another cross-linker, named formaldehyde (FA), is used to detect both direct and indirect interactions between RNA species, which is known as RAP-RNA^[FA]^. Finally, an advanced subtype of RAP-RNA has been developed by using multiple cross-linkers FA and disuccinimidyl glutarate (FA-DSC) which known as RAP-RNA^[FA-DSG]^. It provides a stronger cross-link and it can detect RNA-RNA interactions, even though multiplex protein complexes bound the RNAs [158].

##### Cross-Linking, Ligation and Sequencing of Hybrids (CLASH)

CLASH is an alternative method for mapping RNA-RNA interaction by using UV cross-linking [159,160]. This method is efficient in identifying snoRNA-rRNA and mRNA-miRNA interactions in yeast and human, respectively [159,160]. CLASH has several advantages, such as no protein-protein crosslinked as like chemically cross-linked methods. However, poor ligation rates of RNA and the incapability of IncRNAs interaction investigation are observed [159].

#### 4.3.4. Techniques for Investigating lncRNA-Protein Interaction

##### RNA Immunoprecipitation (RIP)

RIP is the most widely used method to investigate lncRNA-protein interaction in both in vitro and in vivo [163,164]. In principle, RIP is an antibodies-based technique, where RNA-binding protein of interest specific antibodies are employed, and then RNase digestion and lncRNA-protein complex extraction are performed. Finally, cDNA is synthesized by reverse transcription and, subsequently, high-throughput sequencing, such as, NGS is performed, which provides a single based resolution of protein-bound-RNA [164,165,166]. Remarkably, the cross linking process is not obligatory, but it can be used in RIP to capture both direct and indirect bound RNA-protein interactions, respectively [166]. This method has been used to investigate lncRNA-protein interactions. For example, lncRNA-Xist interaction with different proteins, named SUZ12 polycomb repressive complex 2 subunit (Suz12), RbAp48, and embryonic ectoderm development (EeD), was discovered while using the RIP system [177].

##### High-Throughput Sequencing of RNA Isolated by Cross-Linking Immunoprecipitation (HITS-CLIP)

In HITS-CLIP method, UV-mediated crosslinking forms a covalent bond between RNA and amino acids (AAs) [168,169,170]. The CLIP-sequence was first applied. A set of lncRNA bound to RB binding protein 4, chromatin remodeling factor (RBBP4), and enhancer of polycomb homolog 2 (EPC2) proteins was discovered [167]. The limitations of this method include mutation due to the use of UV crosslinking and failure to provide full length transcripts sequence [170].

##### Photoactivatable Ribonucleotide-Enhanced Cross Linking and Immunoprecipitation (PAR-CLIP)

PAR-CLIP is a modified technique from HITS-CLIP with improved crosslinking efficiency and single nucleotide resolution capacity [171,172]. PAR-CLIP utilizes multiple crosslinking agents, such as photoreactive ribonucleotide analogs, 6-thioguanosine (6SG), and 4-thiouridine (4SU), which provide stronger crosslinking efficiency between protein and RNAs. Importantly, the use of these crosslinking reagents also improve the limitations of other methods such as photoreactive ribonucleotide analogs hinders the UV-mediated structural change, 4SU and 6SG improve the sequence specific mutations T to C and G to A, respectively [171,172,173]. This method allows for detecting specific binding sites of RNA-protein complex with higher resolution, but decreased signal-to-noise ratio [86].

## 5. Functional Involvement of lncRNAs in Endothelial Dysfunction-Associated Diseases

**Figure 3:**
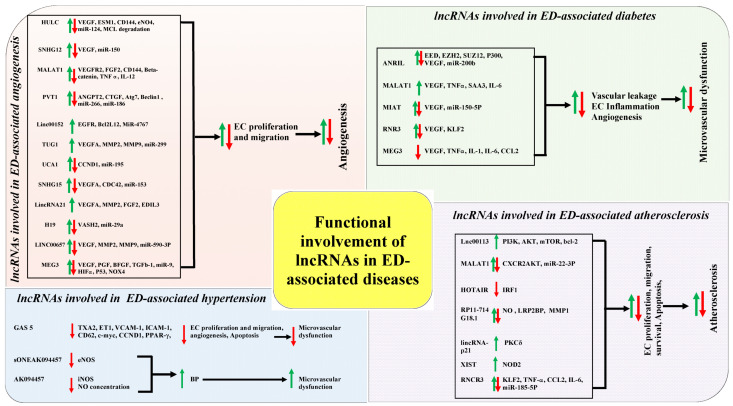
The correlations of functional involvements of long non-coding RNAs (lncRNAs) in abnormal angiogenesis, hypertension, atherosclerosis, and diabetes. Green arrows represent up-regulation and red arrows represent down regulation/ inhibition. All abbreviations mentioned in the figure are spelled out in the end of the article.

lncRNAs have been identified as the key regulators in many biological and pathological processes over the past decades, including impaired endothelial function. A general perspective of the diverse regulatory mechanisms of lncRNAs has been described previously. In this chapter, we closely focus on the functional involvements of lncRNAs in ED-associated diseases, such as abnormal angiogenesis, diabetes, hypertension, and atherosclerosis (Figure 3). It should be reminded that many lncRNAs genes whose expression has positive effects on the expression of other genes might be conferred by the lncRNA gene promoter (ie, enhancer effects in cis) rather than by the lncRNA itself.

### 5.1. The Correlations of Functional Involvement of lncRNAs in ED and Angiogenesis

Angiogenesis is defined as the process of new blood vessel formation from a pre-existing vascular network. The complex process of angiogenesis is predominantly controlled by the precise balance of different stimulating and inhibitory factors, such as VEGFs and their receptors [178]. The dysregulation of angiogenesis process was found to be associated with different life-threating diseases, such as haemangioma and growth restriction of newborn babies, placental insufficiency during pregnancy, cardiovascular disease (CVD), cancer progression and metastasis, and rheumatoid arthritis [179]. It has been revealed that the molecular mechanisms of abnormal angiogenesis are mainly depended on the disease types and conditions. An example is hypoxia regulates sprouting angiogenesis in cancer through multiple pathways [180]. In the hypoxic condition, cancer cells extensively secrete VEGF-A that bind with VEGF receptor 2 (VEGFR2) and subsequently promote tumor angiogenesis through enhanced EC proliferation, increased migration via PI3K/AKT/ mammalian target of rapamycin (mTOR)/mitogen-activated protein kinases (MAPK), and increased nuclear factor kappa-light-chain-enhancer of activated B cells (NFkB) signalling pathways [181]. Another example is the endothelial progenitor cell (EPC), VSMC, and mesenchymal stem cell (MSC) being critically involved in the angiogenesis progression via regulating the expression of different proangiogenic cytokines, growth factors, angiogenic mediators (such as VEGF, TNF-α, IL-1β), and affecting the differentiation efficiency of EC from MSC [182,183].

Over the past couple of decades, extensive researches had utilized the regulatory process of angiogenesis to establish promising therapeutic strategies in complex disease management. For example, strategies for inhibiting angiogenesis were beneficial for cancer, haemangioma, and corneal neovascularization treatment [184]. On the contrary, angiogenesis stimulation was helpful in placental insufficiency, regenerative disorders, and tissue engineering management [185]. More recently, several lncRNAs were found correlated in the regulation of EC functions and angiogenesis gene expression at their transcriptional and post translational levels, and thus possibly intervening the modulation of angiogenesis process and its associated disease development and progression (Table 4).

One of the most common intergenic lncRNAs, named as TUG1, is present on chromosome 22q12.2 and is 7.1 kb in length. It regulates angiogenesis and it can be found in different types of cancers, including glioblastoma and hepatoblastoma progression [69,70,71,194]. TUG1 was highly expressed in hepatoblastoma, which is a common malignant hepatic tumour in children, and it was also associated with an increased risk of tumour metastasis [195,196]. In Dong’s research group study, they demonstrated that TUG1 promotes tumour angiogenesis via up-regulating the expression level of VEGFA by sponging miR-34a [71]. TUG1 is suggested as a potential target to combat the abnormal angiogenesis and improve hepatoblastoma treatment.

Among the angiogenesis promoting lncRNAs, there is one lncRNA, named MEG3, 1.6 kb in length and is present in chromosome 14q32.3 [197], which was found to be negatively correlated with the progression of disease-related angiogenesis. For example, overexpressed MEG3 suppressed breast cancer cell proliferation, invasion, and angiogenesis through the AKT pathway [61]. Similarly, overexpressed MEG3 inhibited osteoarthritis, ischemic brain injury, and cerebral infarction mediated angiogenesis through the alternation of EC functions via regulating the expression levels of VEGF, VEGFR2, notch signalling pathways, and P53/ NADPH Oxidase 4 (NOX4) axis, respectively [62,63,64,65,66]. Taking together, some lncRNAs can be considered as potential targets for regulating EC function and new vessel formation to abate the development and the progression of angiogenesis-related multifaceted diseases.

### 5.2. The Correlations of Functional Involvement of lncRNAs in ED and Diabetes

Diabetes mellitus (DM) is a chronic disorder and it is ranked as the ninth leading cause of the death globally [198]. There is a large amount of evidence showing that both Type-1 DM and Type-2 DM are highly interconnected with ED [199,200,201,202,203]. Hyperglycemia is one of the lethal factors that lead to a reduction in bioavailability of NO and subsequent impaired ED [204,205]. In DM, EC function can be impaired through several mechanisms, such as an alteration in signaling pathways, excessive oxidative stress, activation of pro-inflammatory factors and protein kinase C, and mitochondrial dysfunction [206]. Most recently, several genome-wide studies have been revealed the correlations of the functional involvement of different lncRNAs in ED and diabetes (Table 5) [55,56,57,67,68,75,79,207,208].

One of the most common intergenic lncRNAs, named MALAT1, which is located on chromosome 11q13.1 and 7 kb in size, has been functionally identified and characterized in ED-associated diabetes [55]. In that study, MALAT1 was significantly up-regulated in DM. The knockdown of MALAT1 can alter the retinal EC proliferation, migration, and tube formation through interacting with the p38MAPK signaling pathway [55]. Besides, DM with highly expressed MALAT1 who can upregulate serum amyloid antigen3 (SAA3) and stimulate EC inflammation through inflammatory mediators IL-6 and TNF-α [56,57]. This indicates the upexpressed MALAT1 induced by diabetes might represent an important regulator of EC in DM and it can possibly act as a novel therapeutic target to lessen the burden that is induced by the vascular complication of ED-associated diabetes. Apart from MALAT1, another lncRNA, named MEG3, regulates EC function through activating the PI3K/Akt signaling pathway under hyperglycemic and oxidative stress condition [67,68].

### 5.3. The Correlations of Functional Involvement of lncRNAs in ED and Hypertension

Hypertension is one of the most important risk factors for CVDs. Studies revealed that vascular endothelium nicotinamide adenine dinucleotide phosphate (NADPH) oxidase, excessive production of mitochondrial ROS, and decreased NO bioavailability were intercorrelated with the impairment of EC function [206,210,211,212]. Hypertension associated VEC dysfunction is modulated by both local and systemic inflammation, and it subsequently stimulates the activated complement factor 3 (C3) [213]. Afterwards, the activated C3 augment the activity of EPC and C-reactive protein, which consequently leads to vascular damage [213,214,215]. The number of lncRNA that could be identified in hypertension-associated EC dysfunction is limited when compared to other form of diseases-associated EC dysfunction, but still some lncRNAs have been identified. For example, one of the well-known lncRNAs GAS5, which is located on chromosome 1q25.1 and is widely expressed in different tissues in regulating diverse biological processes, such as cell proliferation, cell growth arrest, and cell apoptosis [36,216,217]. It also regulates vascular remodeling in hypertension [218]. Mechanistically, GAS5 regulates EC and VSMC function in both in vitro and in vivo through interacting with β-cantenin signaling activity [218]. Another study also demonstrated that a traditional Chinese medicine, *L. barbarum*, carries anti-hypertensive effect in both in vitro and in vivo by reducing the blood pressure through up-regulating the expression level of eNOS via suppressing lncRNA sONE, [219]. Similarly, another traditional Asian medicine, named Notoginsenoside R1 (NR1), reduces blood pressure through stimulating the production of NO via increasing the phosphorylation of the inducible NO synthase (iNOS) in EC of the hypertensive animal model. NR1 also down-regulates the overexpressed lncRNA AK094457 in VEC induced the higher expression of iNOS and NO concentration, which leads to the subsequent reduction in blood pressure in hypertensive animal model [220].

### 5.4. The Correlations of Functional Involvement of lncRNAs in ED and Atherosclerosis

Atherosclerosis is a large arteries disorder that is considered to be one of the major causes of CVD [221]. It is generally characterized by the development of atherosclerotic plaques in the arterial intima due to the thickening and loss of artery walls elasticity [222,223]. ED has long been considered as the early marker for atherosclerosis, which can be detected before the onset of structural changes [224]. When the endothelial layer is injured at arterial branches, the decreased eNOS expression and increased NF-KB expression lead to subsequent infiltration of LDLs, leukocytes adhesion, and inflammatory response [225]. The infiltration of LDL enhances the monocytes recruitment at the injured site of arterials walls and stimulates chronic inflammation response, thus inducing atherosclerotic plaques formation [226,227]. Moreover, the activated VSMCs were highly proliferated during impaired function of EC, which leads to the thickening and stiffening of arterial walls [228]. The EC functions might be regulated by diverse lncRNAs and potentially involved in the modulation of different vascular diseases development and progression (Table 6) [229].

An intergenic lncRNA, RNCR3, was found to be significantly up-regulated in both human and mouse aorta atherosclerotic lesion and it accelerates atheroprotective function to the endothelium [78]. In mice models (ApoE−/− and C57BL/6J), the knockdown of RNCR3 promotes atherosclerosis development via aggravated hypercholesterolemia and excessive inflammatory factors release. [78]. Similarly, a lncRNA, named TCONS_00024652, stimulates EC proliferation, migration, and plaque angiogenesis through regulating miR-21 expression, which eventually promotes atherosclerosis progression [233]. All of these established correlations, which suggests potential linkages of the functional involvement of different lncRNAs in ED and atherosclerosis.

## 6. Conclusions

It is recommended to target the aim of lncRNA study at disease specific or cell specific, rather than performing a gross study to discover the biomarkers for CVD due to the complexity of CVS pathophysiology, the interference of IncRNA in gene expression and signal pathways at various stages. Examples of disease specific lncRNAs include MIAT for acute myocardial infarction (AMI) [234] and myosin heavy chain associated RNA transcript (Mhrt) for heart failure [235]. In the vascular system, endothelial expressed MALAT1 can regulate vessel growth and function [59]. On the other hand, SMC-expressed brain cytoplasmic RNA 1 (BCYRN1) mainly regulates its contractile phenotype [236]. In this regard, we believed that systemic tissue specific or condition specific study of lncRNAs can improve their therapeutic potential in the translational perspective.

We also observed that some identified lncRNAs might be playing critical roles in regulating the development and progression of various ED-associated diseases, and therefore possibly to translate into novel biomarkers and therapeutic targets in ED-associated disease treatments. There is a growing body evidence that suggests that ED is functionally associated with various pathophysiological conditions, including abnormal angiogenesis, hypertension, diabetes, and atherosclerosis, which in turns leads to the increased cardiovascular disease-related deaths. Several lncRNAs were correlated in the regulation of endothelial function and its associated diseases progression. However, the underlying functional mechanisms in the majority of these identified lncRNAs are still not clear. Nevertheless, with the discovery of new advanced technologies mentioned earlier, a massive development was underway in lncRNA biology studies and emerging topics, such as largescale discovery of lncRNAs using genome-wide sequencing methods, the identification of lncRNAs subcellular localization, and the potential interactions of lncRNAs with DNA, RNA, and protein. These will help us to better understand about lncRNAs functions in different biological processes, including endothelial dysfunction and its associated disorders.

## Figures and Tables

**Table 1:** An overview of the commonly used methodologies to identify lncRNAs and the advantages and disadvantages over its use. SAGE: Serial analysis of gene expression; CAGE: Cap analysis of gene expression; RNA-seq: RNA sequencing

Method	Advantages	Disadvantages	References
Tiling arrays	Able to identify and quantify the expression level of transcripts (up to few hundred fold)	Limited to distinguish different isoforms and allelic expression Higher background noise and cost effective	[86,88,89]
SAGE	Allows novel transcripts identification Capable to distinguish different isoforms and allelic expression	Relatively low-throughput High cost effective	[90,91]
CAGE	Can identify promoter region and TSS	Limited to the 5′-capped transcripts High cost effective	[92,93,94,95,96]
RNA-Seq	Able to identify and quantify the expression level of transcripts (>8000 fold) Allows to distinguish different isoforms and allelic expression Lower background noise	Time consuming method Complex bioinformatics (data analysis) issues	[97,98,99]

**Table 2:** The advantages and disadvantages of different widely used methods to investigate the cellular functions of lncRNAs in different physiological and/or pathological conditions. RNAi: RNA interference; siRNA: Small interfering RNA; shRNA: Short hairpin RNA; ASOs: Antisense oligonucleotides; sgRNA: Single guide RNA; CRISPRi: CRISPR interference; dCas9: nuclease-dead deactivated Cas9.

Method	Advantages	Disadvantages	References
RNAi	High knockdown efficiency shRNAs allow inducible and sable silencing for long-term Relatively fast and easy to use Cost effective	Inducible and sable long-term silencing is not possible with siRNAs Knockdown variability and incompleteness Off-target effect of siRNA	[112,113,114,115]
ASOs	Able to knockdown nuclear lncRNA Easy to modify probe length to increase efficiency	Less effective to cytoplasmic lncRNA Higher off-target effect	[116,117,118,119]
CRISPR/Cas9	Highly specific and negligible off-target effect High recombination frequency Multiple sgRNA can be used to achieve complete lncRNA perturbation	Interaction with neighboring genes Provides false positive effect	[120,121,122,123,124]
CRISPRi	Provides robust gene suppression and activation Effective in both nucleus and cytoplasmic lncRNA Highly specific with minimal off-target effect	dCas9 can repress downstream genes within an operon instead of an individual gene Multiple components need to transfect into the cell	[125,126]

**Table 3:** Advantages and disadvantages of commonly available methods to identify the subcellular localization and potential interaction of lncRNAs with DNA, RNA, and protein. smRNA-FISH: single-molecule RNA fluorescence in situ hybridization; CHART: Capture hybridization analysis of RNA target; ChIRP: Chromatin isolation by RNA purification; RAP: RNA antisense purification; RAP-RNA: RNA antisense purification followed by RNA sequencing; CLASH: Cross-linking, ligation and sequencing of hybrids; RIP: RNA immunoprecipitation; HITS-CLIP: High-throughput sequencing of RNA isolated by cross-linking immunoprecipitation; PAR-CLIP: Photoactivatable ribonucleotide-enhanced cross linking and immunoprecipitation.

Purpose of Study	Method	Advantages	Disadvantages	References
Identification of subcellular localization	smRNA-FISH	Detect the subcellular localization of low-abundance lncRNA Quantify the absolute level of lncRNA	Possibility to detect false positive result	[149,150]
Investigation of lncRNA-DNA interaction	CHART	No prior knowledge is required Reduced background signal due to fewer probes used	Cross linking agent formaldehyde is not efficient enough Time consuming method	[86,151,152]
	ChIRP	Able to detect genome-wide lncRNA-DNA binding sites Less off-target effect	Incapable to disclose individual lncRNA domain function Higher noise-to-signal ratio	[153,154,155]
	RAP	Able to detect residual lncRNA fragments Provides higher binding affinity to target lncRNA due to larger RNA probe used Reduced signal-to-noise ratio	Difficult to synthesize long probes Higher off-target effect Prior knowledge is required	[156,157]
Investigation of lncRNA-RNA interaction	RAP-RNA	Different versions are available to analyze RNA-RNA interactions Can detect both direct and indirect RNA-RNA interaction	RNA sequence needed to be known Relatively complex to design	[158]
	CLASH	Capable to map RNA-RNA interactions in vivo Provides RNA-RNA binding sites & level resolution	Relatively low efficiency Low ligation rate between short RNA fragments Bait protein information is required	[159,160,161,162]
Investigation of lncRNA-protein interaction	RIP	Capable to map specific RNA-Protein complexes Higher binding resolution and low background signal No prior knowledge is required	Required known protein specific antibodies False negative result can be generated Nonspecific antibodies cannot be used	[163,164,165,166]
	HITS-CLIP	Reduced background noise Able to detect actual RBP binding sites even with few nucleotides	Low sensitivity compared to RIP Mutations can be induced due to the use of UV light	[167,168,169,170]
	PAR-CLIP	Provides efficient mapping of RNA-protein interaction Decreased signal-to-noise ratio Develop specific sequence mutations	Limited to in vitro study Pull-down of non-specific protein	[171,172,173]

**Table 4:** The correlations of the functional involvement of different lncRNAs in ED and angiogenesis. HULC: Highly upregulated in liver cancer; ESM-1: endothelial cell specific molecule; PI3K: Phosphatidylinositol 3-kinase; Akt: AKT serine/threonine kinase 1; EC: Endothelial cell; SNHG12: Small nucleolar RNA host gene 12; OGD: oxygen-glucose deprivation; VEGF: Vascular endothelial growth factor; MALAT1: Metastasis associated lung adenocarcinoma transcript 1; SMMECs: Skeletal muscle microvascular endothelial cells; VEGFR2: VEGF receptor 2; TAMs: Tumor-associated macrophages; ERK: extracellular signal-regulated kinase; MMP: Matrix metalloproteinase; FAK: Focal adhesion kinase; FGF2: Fibroblast Growth Factor 2; UBE2CP3: Ubiquitin conjugating enzyme E2 C pseudogene 3; VEGFA: Vascular endothelial growth factor A; ERK1: Extracellular signal-regulated kinase 1; HIF-1α: hypoxia inducible factor 1 subunit alpha; PVT1: Plasmacytoma variant translocation 1; CTGF: connective tissue growth factor; ANGPT2: angiopoietin 2; HUVECs: Human umbilical vein endothelial cells; VEC: vascular endothelial cells; oxLDL: oxidized low-density lipoprotein; TUG1: Taurine up-regulated 1; UCA1: Urothelial cancer associated 1; HMEC: Human microvascular endothelial cells; MEK: Mitogen-activated protein kinase kinase; MEG3: Maternally expressed 3; RBMVECs; Rat brain microvascular endothelial cells; NOX4: NADPH Oxidase 4; Cdc42: cell division cycle 42; HOTAIR: HOX transcript antisense RNA; GRP78: glucose regulated protein 78.

Name	Location	Disease Association	Correlations of the Functional Involvement of lncRNAs in ED and Angiogenesis	References
HULC	6p24.3	Gliomas	Promotes angiogenesis through regulating endothelial cell specific molecule 1 (ESM-1) via PI3K/AKT/mTOR pathway	[49]
		Angiogenesis	Regulates EC angiogenesis via sequestrating miR-124	[50]
Small nucleolar RNA host gene 12 (SNHG12)	1p35.3	Ischemic stroke	Promotes angiogenesis of oxygen-glucose deprivation (OGD)-treated brain microvascular EC through regulating miR-150/VEGF pathway	[186]
MALAT1	11q13.1	Hindlimb ischemia	Promotes cell-autonomous angiogenesis of skeletal muscle microvascular endothelial cells (SMMECs) via VEGFR2 regulation	[51]
		Thyroid cancer	Promotes angiogenesis through regulating fibroblast Growth Factor 2 (FGF2) protein secretion from tumor-associated macrophages (TMAs)	[52]
		Gastric cancer	Correlated with endothelial vessel density and promotes angiogenesis by regulating VE-cadherin/β-catenin complex, extracellular signal-regulated kinase (ERK)/ matrix metalloproteinase (MMP), and focal adhesion kinase (FAK)/paxillin signalizing pathways	[53]
		Neuroblastoma	Promotes hypoxia-induced angiogenesis by increasing FGF2 expression	[54]
Ubiquitin conjugating enzyme E2 C pseudogene 3 (UBE2CP3)	4q12	Hepatocellular carcinoma	Stimulates vascular endothelial growth factor A (VEGFA) secretion and promotes HUVEC proliferation, migration and tube formation through activating ERK1/2/ hypoxia inducible factor 1 subunit alpha (HIF-1α)/VEGFA signalling pathways	[187]
PVT1	8q24.21	Angiogenesis	Regulates connective tissue growth factor (CTGF) and angiopoietin 2 (ANGPT2) expression through interacting with miR-26b and promotes the angiogenesis of HUVEC	[72]
		Glioma	Promotes glioma VEC proliferation, migration and angiogenesis by regulating miR-186	[73]
Linc00152	2p11.2	Angiogenesis	Promotes oxidized low-density lipoprotein (oxLDL)-treated HUVEC migration and inhibits apoptosis by sponging miR-4767 that regulates angiogenesis	[188]
TUG1	22q12.2	Glioblastoma	Promotes glioblastoma-induced EC proliferation, migration and angiogenesis by inhibiting miR-299	[69]
		Angiogenesis	Regulates rapamycin-mediated inhibition of EC proliferation, migration and tube formation	[70]
		Hepatoblastoma	Promotes angiogenesis via regulating VEGFA expression by sponging miR-34a	[71]
Urothelial cancer associated 1 (UCA1)	19p13.12	Angiogenesis	Promotes human microvascular endothelial cells (HMEC) proliferation, migration and tube formation through inhibiting miR-195 and activating mitogen-activated protein kinase kinase (MEK)/ERK/mTOR pathways	[189]
MEG3	14q32.2	Breast cancer	Overexpressed MEG3 suppresses tumour growth and angiogenesis via inhibiting AKT pathway	[61]
		Osteoarthritis	Regulates angiogenesis through inversely association of VEGF levels	[62]
		Angiogenesis	Overexpressed MEG3 suppresses VEC proliferation, migration and angiogenesis via regulating miR-9	[63]
		Ischemic brain injury	Downregulated MEG3 promotes angiogenesis via negatively regulated Notch pathways	[64]
		Cerebral infarction	Downregulated MEG3 promotes angiogenesis of OGD/R-induced rat brain microvascular endothelial cells (RBMVECs) through regulating P53/NOX4 axis	[65]
		Angiogenesis	Downregulated MEG3 suppresses VEGF-induced EC migration and angiogenesis through modulating VEGFR2 expression	[66]
Linc00511	17q24.3	Pancreatic ductal adenocarcinoma	Promotes tumour cells proliferation, migration, invasion and angiogenesis through sponging miR-29b-3p	[190]
Small nucleolar RNA host gene 15 (SNHG15)	7p13	Glioma	Promotes glioma VEC proliferation, migration and tube formation by increasing VEGFA and cell division cycle 42 (Cdc42) expression by targeting miR-153	[191]
LincRNA-p21	6p21.2	Non–Small Cell Lung Cancer	Enhances VEGFA production and promotes angiogenesis	[192]
ANRIL	9p21.3	Diabetes mellitus	Overexpressed ANRIL promotes angiogenesis by increasing VEGF expression via activating NF-kB pathway	[74]
HOTAIR	12q13.13	Nasopharyngeal carcinoma	Promotes angiogenesis through upregulating VEGFA and angiopoietin 2 (Ang2) expression by glucose regulated protein 78 (GRP78).	[76]
H19	11p15.5	Glioma	Promotes glioma-induced angiogenesis by increasing miR-29a	[81]
LINC00657	20q11.23	Angiogenesis	Promotes oxLDL-mediated EC proliferation, migration and tube formation by interacting with miR-590-3p and increasing VEGF, MMP-2 and MMP-9 expression	[193]

**Table 5:** The correlations of the functional involvement of different lncRNAs in ED and diabetes. DM: Diabetes mellitus; IL-6: interleukin-6; TNF-α: Tumor necrosis factor-α; SAA3: Serum amyloid antigen3; MAPK: mitogen-activated protein kinases; PI3K: Phosphatidylinositol 3-kinase; Akt: AKT serine/threonine kinase 1; CeRNA: competing endogenous RNA; MIAT: Myocardial infarction associated transcript; RNCR3: Retinal non-coding RNA3; KLF2: Kruppel like factor 2.

lncRNA	Correlations of the Functional Involvement of lncRNAs in ED and Diabetes	References
ANRIL	Up-regulated in DM and alters the EC function through increasing VEGF expression by P300/miR200b/EZH2	[75]
MALAT1	Highly expressed in DM and upregulates inflammatory mediators IL-6 & TNF-α through activating SAA3 and that stimulates DM-induced EC dysfunction via p38MAPK signaling pathway	[55,56,57]
MEG3	Down-expressed in DM and enhances DM-mediated EC dysfunctions through altering PI3K/Akt signaling pathway	[67,68]
Myocardial infarction associated transcript (MIAT)	Induces DM induced EC dysfunction by acting as a competing endogenous RNA (CeRNA) via MIAT/miR-150-5p/VEGF network	[209]
RNCR3	Up-regulated in DM and stimulates DM-induced retinal EC dysfunction through regulating RNCR3/ Kruppel like factor 2 (KLF2)/miR-185-5p pathway	[79]

**Table 6:** The correlations of the functional involvement of different lncRNAs in ED and atherosclerosis. EC: Endothelial cells; PI3K: Phosphatidylinositol 3-kinase; Akt: AKT serine/threonine kinase 1; mTOR: Mammalian target of rapamycin; MALAT1: Metastasis associated lung adenocarcinoma transcript 1; oxLDL: oxidized low-density lipoprotein; CXCR2: C-X-C Motif Chemokine Receptor 2; HOTAIR: HOX transcript antisense RNA; TSLP: thymic stromal lymphopoietin; LRP2BP: LRP2 binding protein; MMP1: matrix metallopeptidase 1; LOX-1: Lysyl oxidase-like 1; PKCδ; protein kinase C delta; NOD2: nucleotide binding oligomerization domain containing 2; CeRNA: competing endogenous RNA; KLF2: Kruppel like factor.

lncRNA.	Correlations of the Functional Involvement of lncRNAs in ED and Atherosclerosis	References
Lnc00113	Promotes abnormal EC proliferation, survival and migration via activating PI3K/Akt/mTOR pathway that disrupt EC function and develop atherosclerosis	[230]
MALAT1	Protects EC from ox-LDL-induced EC dysfunction through inhibiting miR-22-3P and upregulating C-X-C Motif Chemokine Receptor 2 (CXCR2) & AKT expression in the settings of atherosclerosis	[58]
HOTAIR	Protect EC from injury and enhance EC proliferation, migration and inhibit apoptosis via thymic stromal lymphopoietin (TSLP)-PI3K/AKT-HOTAIR pathway that regulates angiogenesis pathogenesis and progression.	[77]
RP11-714G18.1	Suppresses EC migration through RP11-714G18.1/ *LRP2 binding protein* (LRP2BP)/MMP1 signaling pathway that provide athero-defensive role in atherosclerosis-related EC dysfunction.	[231]
lincRNA-p21	Stimulates ox-LDL-induced EC apoptosis and LOX-1 expression via activation of protein kinase C delta (PKCδ) that regulates atherosclerosis pathogenesis	[232]
TCONS_00024652	Promotes EC proliferation, migration and angiogenesis via downregulating miR21 expression that stimulates atherosclerosis progression	[233]
XIST	Promotes ox-LDL-mediated EC injury via miR-320/ nucleotide binding oligomerization domain containing 2 (NOD2) pathway and modulates atherosclerosis	[80]
RNCR3	Regulates EC function and accelerates atheroprotective function to the endothelium via acting as a ceRNA and forming a feedback loop with KLF2 and miR-185-5p	[78]

## Abbreviations

6SG	6-Thioguanosine
4SU	4-Thiouridine
AAs	Amino acids
ADAR	Adenosine deaminase acting on RNA
Akt	AKT serine/threonine kinase 1
Alu	Arthrobacter luteus
AMT	Aminomethyltrioxalen
ANGPT2	Angiopoietin 2
ASOs	Antisense oligonucleotides
BACE1	Beta-secretase 1
BACE1AS	BACE1 (beta-secretase 1)-antisense RNA
BCYRN1	Brain cytoplasmic RNA 1
BP	Blood pressure
CAGE	Cap analysis of gene expression
CCNA2	Cyclin A2
CCNB1	Cyclin B1
CCNB2	Cyclin B2
Cdc42	Cell division cycle 42
CeRNA	Competing endogenous RNA
cGMP	Cyclic guanosine 3′, 5′-monophosphate
CHART	Capture hybridization analysis of RNA target
ChIRP	Chromatin isolation by RNA purification
CLASH	Cross-linking, ligation and sequencing of hybrids
COUP-TFII	Chicken ovalbumin upstream promoter transcription-factor-2
CRISPRi	CRISPR interference
CTD	Carboxyl-teminal domain
CTGF	Connective tissue growth factor
CVD	Cardiovascular disease
CX3CL1	C-X3-C motif chemokine ligand 1
DSC	Disuccinimidyl glutarate
EC	Endothelial cell
ED	Endothelial dysfunction
EDHF	Endothelium derived hyperpolarizing factor
EeD	Embryonic ectoderm development
ERK	Extracellular signal-regulated kinase
ESM-1	Endothelial cell specific molecule
EPC	Endothelial progenitor cell
EPC2	Enhancer of polycomb homolog 2
ER	Endoplasmic reticulum
ET-1	Endothelin-1
FA	Formaldehyde
FAK	Focal adhesion kinase
FISH	Fluorescence in situ hybridization
FGF2	Fibroblast Growth Factor 2
GAS5	Growth arrest specific 5
GATA-AS	Antisense transcript of GATA6
GRP78	Glucose regulated protein 78
GR	Glucocorticoid receptor
GTP	Guanosine triphosphate
H3K4me2	Demethylation of lysine 4 residue on histone 3
H3K4me3	Trimethylation of lysine 4 of histone H3
H3K27me3	Histone 3 lysine 27 trimethylation
H19	H19 imprinted maternally expressed transcript
HDR	Homology direct repair
HITS-CLIP	High-throughput sequencing of RNA isolated by cross-linking immunoprecipitation
HOTAIR	HOX transcript antisense RNA
HULC	Highly upregulated in liver cancer
IL-6	Interleukin-6
HUVECs	Human umbilical vein endothelial cells
IL-1β	Interleukin 1 beta
KLF2	Kruppel like factor
KRAB	Kruppel-associated box
LDL	Low-density lipoprotein
lncRNAs	Long non-coding RNAs
LOX2	Lysyl oxidase-like 2
LRP2BP	LRP2 binding protein
LSD1	Lys-specific demethylase 1
MALAT1	Metastasis associated lung adenocarcinoma transcript 1
MAPK	Mitogen-activated protein kinases
MCP-1	Monocyte chemoattractant protein-1
MEG3	Maternally expressed 3
MERFISH	Multiplexed error-robust fluorescence in situ hybridization
Mhrt	Myosin heavy chain associated RNA transcript
MIAT	Myocardial infarction associated transcript
MMP	Matrix metalloproteinase
MSC	Mesenchymal stem cell
mTOR	Mammalian target of rapamycin
NADPH	Nicotinamide adenine dinucleotide phosphate
NATs	Natural antisense transcripts
NFAT	Nuclear factor of activated T-cells
NFkB	Nuclear factor kappa-light-chain-enhancer of activated B cells
NGS	Next generation sequencing
NHEJ	Non-homologous end joining
NO	Nitric oxide
NOD2	Nucleotide binding oligomerization domain containing 2
eNOS	NO synthase
NR1	Notoginsenoside R1
NRON	Non-coding repressor of NFAT
NOX4	NADPH Oxidase 4
OGD	Oxygen-glucose deprivation
ORF	Open reading frame
oxLDL	Oxidized low-density lipoprotein
PAR-CLIP	Photoactivatable ribonucleotide-enhanced cross linking and immunoprecipitation
PGI2	Prostacyclin
PGH2	ProstaglandinH2
PI3K	Phosphatidylinositol 3-kinase
PKCδ	Protein kinase C delta
PolII	RNA polymerase II
PRC2	Polycomb repressive complex
PVT1	Plasmacytoma variant translocation 1
RAP	RNA antisense purification
RAP-RNA	RNA antisense purification followed by RNA sequencing
RBBP4	RB binding protein 4, chromatin remodeling factor
RIP	RNA immunoprecipitation
RNA-FISH	RNA fluorescence in situ hybridization
RNA-Seq	RNA sequencing
RNAi	RNA interference
RNCR3	Retinal non-coding RNA3
ROS	Reactive oxygen species
SAGE	Serial analysis of gene expression
SAA3	Serum amyloid antigen3
sGC	Soluble guanylyl cyclase
sgRNA	Single guide RNA
shRNA	Short hairpin RNA
SINE	Short interspersed elements
siRNA	Small interfering RNA
SMAD6	Mothers against decapentaplegic homologue 6
smRNA-FISH	Single molecule RNA in situ hybridization
SMMECs	Skeletal muscle microvascular endothelial cells
SNHG12	Small nucleolar RNA host gene 12
SNHG15	Small nucleolar RNA host gene 15
SOX18	SRY (Sex Determining Region Y)-Box 18
SRY	Sex Determining Region Y
Suz12	SUZ12 polycomb repressive complex 2 subunit
T1DM	Type 1 Diabetic Mellitus
T2DM	Type 2 Diabetic Mellitus
TAMs	Tumor-associated macrophages
TSLP	Thymic stromal lymphopoietin
TNF-α	Tumor necrosis factor-α
TSS	Transcriptional start site
TUG1	Taurine up-regulated 1
TXA2	Thromboxane A2
UBE2CP3	Ubiquitin conjugating enzyme E2 C pseudogene 3
UCA-1	Urothelial cancer associated 1
VCAM-1	Vascular cell adhesion molecule-1
VEC	vascular endothelial cells
VSMC	Vascular smooth muscle cells
VECs	Vascular endothelial cells
VEGF	Vascular endothelial growth factor
VEGFA	Vascular endothelial growth factor A
VEGFR2	VEGF receptor 2
XIST	X-inactive specific transcript.
